# Impact of circadian rhythm change on postoperative delirium in elderly patients undergoing lower extremity orthopedic surgery: a propensity score-matched prospective cohort study

**DOI:** 10.3389/fmed.2026.1732335

**Published:** 2026-02-11

**Authors:** Feng Xiao, Qing Zhong, Hui Liu, Zehao Liu, Funing Liu, Yunqiong Zhang, Ying Zhang

**Affiliations:** 1 Department of Anesthesiology, The Affiliated Traditional Chinese Medicine Hospital, Southwest Medical University, Luzhou, China; 2 Luzhou Hospital of Traditional Chinese Medicine, Southwest Medical University, Luzhou, China; 3 Department of Anesthesiology, The People’s Hospital of Jianyang City, Southwest Medical University, Chengdu, Sichuan, China; 4 Luzhou Key Laboratory of Research for Integrative on Pain and Perioperative Organ Protection, Luzhou, Sichuan, China

**Keywords:** circadian rhythm, elderly patients, lower limb fracture, postoperative delirium, propensity score matching

## Abstract

**Background:**

Postoperative delirium (POD) is a common and clinically significant complication associated with numerous adverse outcomes, including prolonged hospitalization, functional decline, and increased mortality. Older adults undergoing lower extremity orthopedic surgery constitute a particularly high-risk population for POD. Previous studies have reported an incidence of POD as high as 34.2% among elderly patients undergoing hip surgery receiving general anesthesia. Although some research has examined the influence of circadian rhythm on cognitive function, the comparative impact of perioperative circadian rhythm change on POD incidence in elderly patients undergoing lower extremity orthopedic procedures remains unexplored. This study therefore undertakes a prospective cohort design to investigate the association between perioperative circadian rhythm change and the risk of POD in this vulnerable patient group.

**Methods:**

Eligible participants were patients aged ≥60 years, of any gender, with American Society of Anesthesiologists (ASA) physical status classification I–III, who underwent elective lower extremity orthopedic surgery under general anesthesia at our institution. Patients were categorized into two groups based on perioperative stability of circadian rhythm: the circadian rhythm change group (Group C) and the no circadian rhythm change group (Group NC). To minimize baseline imbalances between groups, 1:1 propensity score matching (PSM) was performed using a nearest-neighbor algorithm with a caliper width of 0.05. Following matching, multivariable logistic regression was used to assess the association between circadian rhythm change and the incidence of postoperative delirium (POD), while linear regression was employed to evaluate its effect on length of hospital stay. Subgroup analyses were further conducted to explore potential effect modification and to address residual confounding within the matched cohort.

**Results:**

A total of 277 patients met the inclusion criteria and were included in the analysis between August 2024 and January 2025. Following propensity score matching, POD occurred in 37 patients (34.58%) in the Group C and 20 patients (18.69%) in the Group NC. After identifying independent variables potentially associated with POD and adjusting for confounders using binary logistic regression, we found that perioperative circadian rhythm change was independently associated with a higher risk of POD (OR = 2.26; *p* = 0.02). Additionally, multiple linear regression analysis revealed that circadian rhythm change was significantly associated with a longer hospital length of stay. Subgroup analyses suggested a stronger association between circadian rhythm change and POD in patients <75 years (OR = 5.82, *p* = 0.001) and those with better postoperative sleep quality (PSQI ≤8; OR = 6.09, *p* < 0.001), though no interaction remained significant after Bonferroni correction.

**Conclusion:**

Perioperative circadian rhythm change—indexed by MEQ-SA score shifts—is an independent risk factor for postoperative delirium and prolonged hospitalization in older adults undergoing lower extremity orthopedic surgery, highlighting circadian stability as a novel, modifiable target for perioperative neuroprotection.

## Introduction

The global aging population has led to a steady increase in surgical interventions among older adults, particularly those undergoing lower extremity orthopedic procedures due to osteoporosis and age-related declines in physical coordination. Postoperative delirium (POD)—an acute neurocognitive disorder characterized by fluctuating attention, cognitive disturbances, altered level of consciousness, and disruption of circadian rhythm—is a common and clinically significant complication in this vulnerable population. Previous studies have reported an incidence of POD as high as 34.2% among elderly patients undergoing hip surgery receiving general anesthesia (1). Importantly, POD often manifests independently of preexisting psychiatric disorders or chronic use of psychotropic medications. The peak incidence of postoperative delirium (POD) typically occurs within the first postoperative week (days 1–7), although symptoms may persist for up to 30 days in some patients (2). Emerging evidence highlights circadian rhythm change as a potential mechanistic contributor to POD development (3). The suprachiasmatic nucleus (SCN)-regulated circadian system governs critical physiological processes, including sleep–wake cycles, hormonal secretion, and inflammatory responses. Nocturnal melatonin suppression—a reliable biomarker of circadian function—correlates strongly with postoperative sleep disturbances and has been implicated in the pathogenesis of POD (4–6). Mechanistically, circadian rhythm change may exacerbate neuroendocrine dysfunction, oxidative stress, and impaired glymphatic clearance, collectively increasing susceptibility to delirium (7–9). Therapeutic interventions targeting circadian restoration—such as light therapy, melatonin supplementation, and sleep hygiene optimization—show promise in mitigating POD risk (10–12), though robust clinical evidence specifically in elderly populations remains limited. This investigation examines perioperative circadian rhythm change as a modifiable risk factor for POD in older adults undergoing lower extremity orthopedic surgery, with the aim of informing preventive strategies that enhance postoperative cognitive recovery and functional outcomes. We hypothesized that perioperative circadian rhythm change is independently associated with increased risk of POD in elderly patients undergoing lower extremity orthopedic surgery.

## Materials and methods

### Patient recruitment and ethics

This single-center, prospective, observational cohort study was conducted at The People’s Hospital of Jianyang City between August 2024 and January 2025. The protocol was reviewed and approved by the Clinical Research Ethics Committee of The People’s Hospital of Jianyang City (Ethics Approval No. JY20241021X). The study was prospectively registered in the Chinese Clinical Trial Registry (ChiCTR2400087955; http://www.chictr.org.cn) on August 7, 2024, and participant enrollment commenced on August 8, 2024, in compliance with ICMJE requirements for prospective trial registration. All procedures adhered to the ethical principles of the Declaration of Helsinki. Prior to enrollment, researchers screened patients for eligibility based on predefined inclusion and exclusion criteria. Written informed consent was obtained directly from all participants, as they were cognitively intact and capable of providing autonomous consent prior to surgery. A total of 293 eligible patients undergoing lower extremity orthopedic surgery were enrolled and classified into two groups according to the presence or absence of perioperative circadian rhythm change: Group C and Group NC. This report was prepared in accordance with the Strengthening the Reporting of Observational Studies in Epidemiology (STROBE) cohort checklist (13).

### Participants

Our inclusion criteria are as follows: (1) Patients scheduled for elective surgery aged 60 or above; (2) ASA (American Society of Anesthesiologists) physical status grade I–III; (3) Elective lower extremity orthopedic surgery under general anesthesia. Those who met any of the following criteria were excluded: (1) Preoperative delirium; (2) With chronic pain and preoperative neurological disease; (3) Preoperative is calm, anti-depressant or anti-anxiety medications; (4) History of cognitive dysfunction; (5) Always abuse of opioid, nonsteroidal, or other analgesic drug history; (6) Left ventricular ejection fraction <40% of the cardiac insufficiency; (7) Severe renal insufficiency (EGFR <30 mL/min/1.73 m^2^); (8) Refused to give informed consent.

### Anesthesia protocol

Radial artery puncture and catheterization were performed under local anesthesia to enable continuous invasive arterial blood pressure monitoring. Ultrasound-guided regional nerve blockade was administered according to the surgical site, using 0.25% ropivacaine at a dose of 1 mg/kg. General anesthesia was induced via a stepwise intravenous regimen: midazolam (0.04 mg/kg), sufentanil (0.2–0.3 μg/kg), etomidate (1.0–2.0 mg/kg), and cisatracurium (0.1–0.15 mg/kg) were administered sequentially. After 3 min of preoxygenation, tracheal intubation was performed. Mechanical ventilation was managed using a lung-protective strategy: tidal volume of 6–8 mL/kg based on ideal body weight, positive end-expiratory pressure (PEEP) of 3–5 cmH₂O, respiratory rate of 12–14 breaths/min, and an inspiratory-to-expiratory (I:E) ratio of 1:2. Arterial partial pressure of carbon dioxide (PaCO₂) was maintained within 40–45 mmHg. Anesthesia was maintained with a balanced technique combining intravenous and inhalational agents: continuous infusion of propofol (1.0–1.5 mg/kg/h) and remifentanil (0.2–0.5 μg/kg/min), supplemented with sevoflurane (1–1.5% end-tidal concentration). Additional cisatracurium was administered as needed to maintain neuromuscular blockade. Depth of anesthesia was monitored using the bispectral index (BIS), with values maintained between 40 and 60 throughout the procedure. Hemodynamic stability was preserved by titrating vasoactive agents (e.g., norepinephrine, esmolol) to keep systolic blood pressure and heart rate within ±20% of baseline values. At the conclusion of surgery, all anesthetic agents were discontinued, and patients were transferred to either the post-anesthesia care unit (PACU) or the intensive care unit (ICU) based on clinical status. In the PACU, standardized reversal protocols were applied: neostigmine (0.015 mg/kg) with glycopyrrolate to antagonize residual neuromuscular blockade, naloxone (10 μg) to reverse opioid-induced respiratory depression, and flumazenil (0.2 mg) to reverse benzodiazepine effects. Postoperative analgesia was provided via patient-controlled intravenous analgesia (PCIA) consisting of sufentanil (2–3 μg/kg), dexmedetomidine (10 μg/kg), and ramosetron (15 mg), diluted to a total volume of 120 mL with 0.9% saline. The background infusion rate was set at 1.5 mL/h, patients were instructed to press the bolus button to manage breakthrough pain during nursing care, dressing changes, or mobilization (12).

### Sample size calculation

According to the literature (14) and previous pre-experiments, the incidence of delirium in group C was 38%, and that in group NC was 20%. Assuming bilateral *α* = 0.05 and the success rate was 80%. The sample size was calculated using PASS15 software, considering the situations of loss to follow-up and refusal to visit (10%). The results showed that at least 212 patients were needed.

### Data collection and outcome measures

We collected: (1) preoperative baseline data (sex, age, BMI, ASA grade, education, and comorbidities including coronary heart disease, hypertension, diabetes, smoking, and alcohol use); (2) preoperative and early postoperative (within 72 h) biochemical markers (serum albumin, sodium, creatinine, glucose, CRP, white blood cell count, hemoglobin, and platelets); and (3) intraoperative variables (anesthesia start time, total doses of anesthetics—sufentanil, cisatracurium, propofol, sevoflurane, remifentanil—estimated blood loss, transfusion, fluid administration, urine output, anesthesia duration, ICU stay, and postoperative analgesia).

The primary outcome was POD within 5 days; the secondary outcome was hospital length of stay. Sleep-related measures included pre- and PSQI scores, daily total sleep duration, nap duration, sleep midpoint, phase shift, outdoor daylight exposure, and iatrogenic sleep disruptions during the first five postoperative nights (derived from nursing records, the number of iatrogenic sleep disruption was used as a surrogate indicator of ward-related environmental disruption).

Circadian rhythm was assessed using the MEQ-SA preoperatively and on postoperative morning day 1. A change in MEQ-SA classification (evening, intermediate-evening, intermediate, intermediate-morning, or morning type) defined “circadian rhythm change.” POD was evaluated twice daily (08:00 and 20:00) on postoperative days 1, 3, and 5 using the Chinese version of the 3-Minute Diagnostic Interview for Confusion Assessment Method-Defined Delirium (3D-CAM-CN) in general wards or ICU-CAM in the ICU; diagnosis required features 1 (acute onset/fluctuation) and 2 (inattention), plus either feature 3 (altered consciousness) or 4 (disorganized thinking) (15, 16). All assessments (MEQ-SA, PSQI, VAS for resting pain) were administered only when patients were delirium-free; otherwise, they were postponed until delirium resolved, with reassessment every 2 h. Two trained physicians independently conducted all questionnaire evaluations. Written informed consent was obtained from patients and their families prior to enrollment.

### Statistical analysis

The analysis was conducted using SPSS version 25.0 (IBM Corp., Armonk, NY, United States). In this study, propensity score matching (PSM) was performed using a nearest-neighbor algorithm with a caliper width of 0.05 to generate a 1:1 matched cohort. After PSM, the distribution of variables was assessed to guide the choice of statistical methods. For continuous variables that followed a normal distribution, descriptive statistics were presented as mean ± standard deviation (mean ± SD), and between-group comparisons were performed using the independent samples *t*-test. For continuous variables with a non-normal distribution, data were summarized as median (interquartile range) [M (IQR)], and the Mann–Whitney *U* test was used for univariate analysis. Categorical variables were expressed as frequencies (*n*) and percentages (%). Between-group comparisons for categorical variables were conducted using the chi-square test or Fisher’s exact test, depending on sample size and expected cell frequencies. A two-sided significance level of *α* = 0.05 was used to determine statistical significance. Variables that achieved *p* < 0.05 in univariate analyses were subsequently included in multivariable regression models, with binary logistic regression employed for binary outcomes (e.g., POD occurrence) and multiple linear regression used for continuous outcomes (e.g., length of hospital stay). Given the observational nature of this study, subgroup analyses were conducted in an exploratory fashion to generate hypotheses. To mitigate the risk of false-positive findings due to multiple comparisons, we applied the Bonferroni method to adjust the interaction *p* values across all selected subgroups.


$$n=\frac{{\left(Z_{\alpha /2}\sqrt{2\bar{p}(1–\bar{p})}+Z_{\beta }\sqrt{p_{1}(1–p_{1})+p_{2}(1–p_{2})}\right)}^{2}}{{\left(p_{1}–p_{2}\right)}^{2}}$$

## Results

### Patient screening and cohort assembly

**Figure 1:**
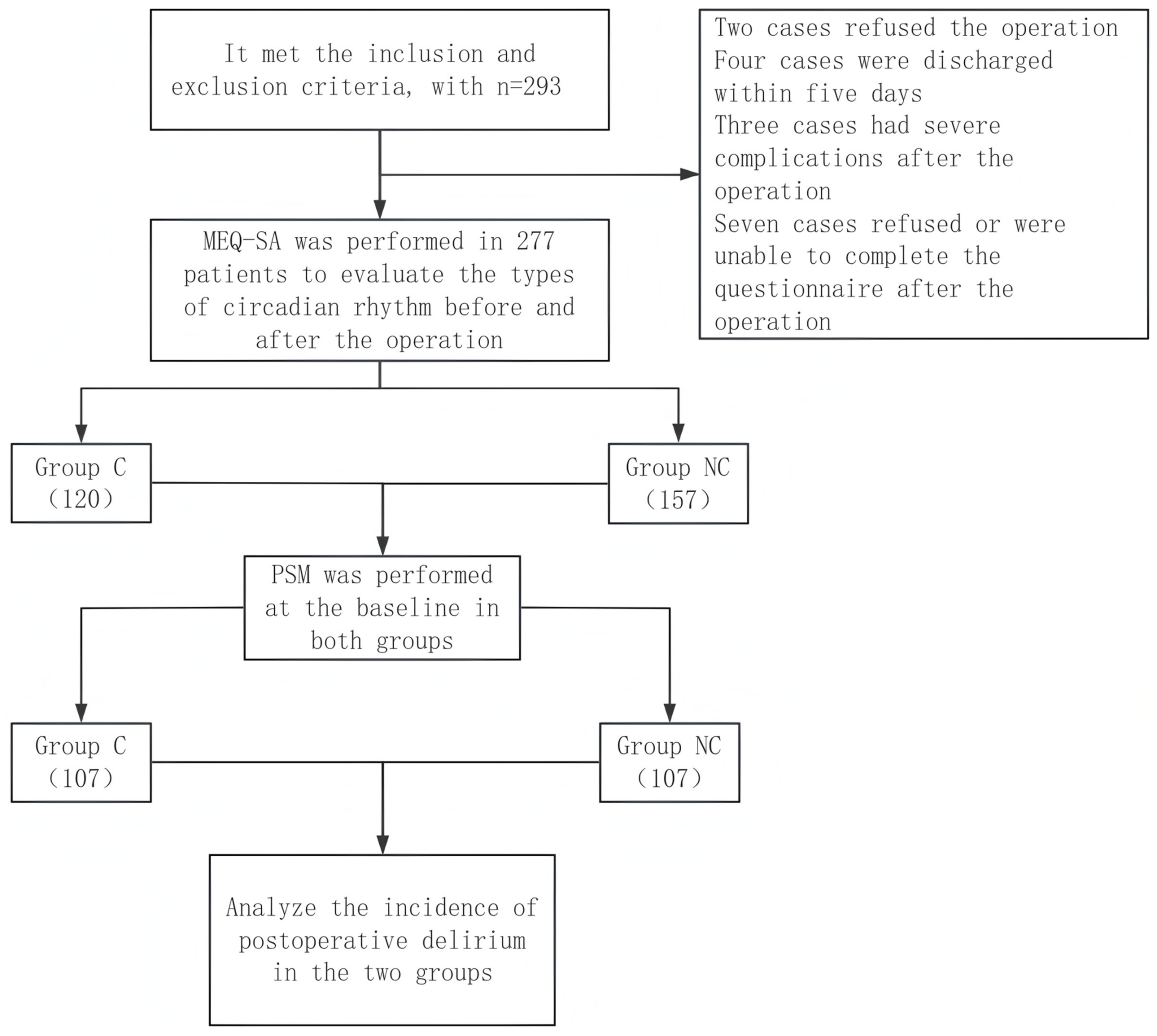
CONSORT flow diagram summarizing study participant disposition.

Of the 293 patients initially screened, 277 were included in the final analysis after excluding 16 individuals due to refusal of surgery (*n* = 2), early discharge (*n* = 4), severe postoperative complications precluding assessment (*n* = 3), or incomplete circadian questionnaires (*n* = 7) (Figure 1). Among the analytic cohort, 120 patients exhibited significant perioperative circadian rhythm change (Group C) and 157 did not (Group NC).

### Propensity score matching

To minimize confounding by baseline differences, we performed 1:1 nearest-neighbor propensity score matching without replacement. The propensity score was estimated using a logistic regression model incorporating age, sex, BMI, education level, ASA physical status class, comorbidities (hypertension, diabetes, coronary heart disease), lifestyle factors (smoking, alcohol consumption), and surgical site. After matching, 107 well-matched pairs were retained for subsequent analyses.

### Assessment of baseline balance after matching

**Figure 2:**
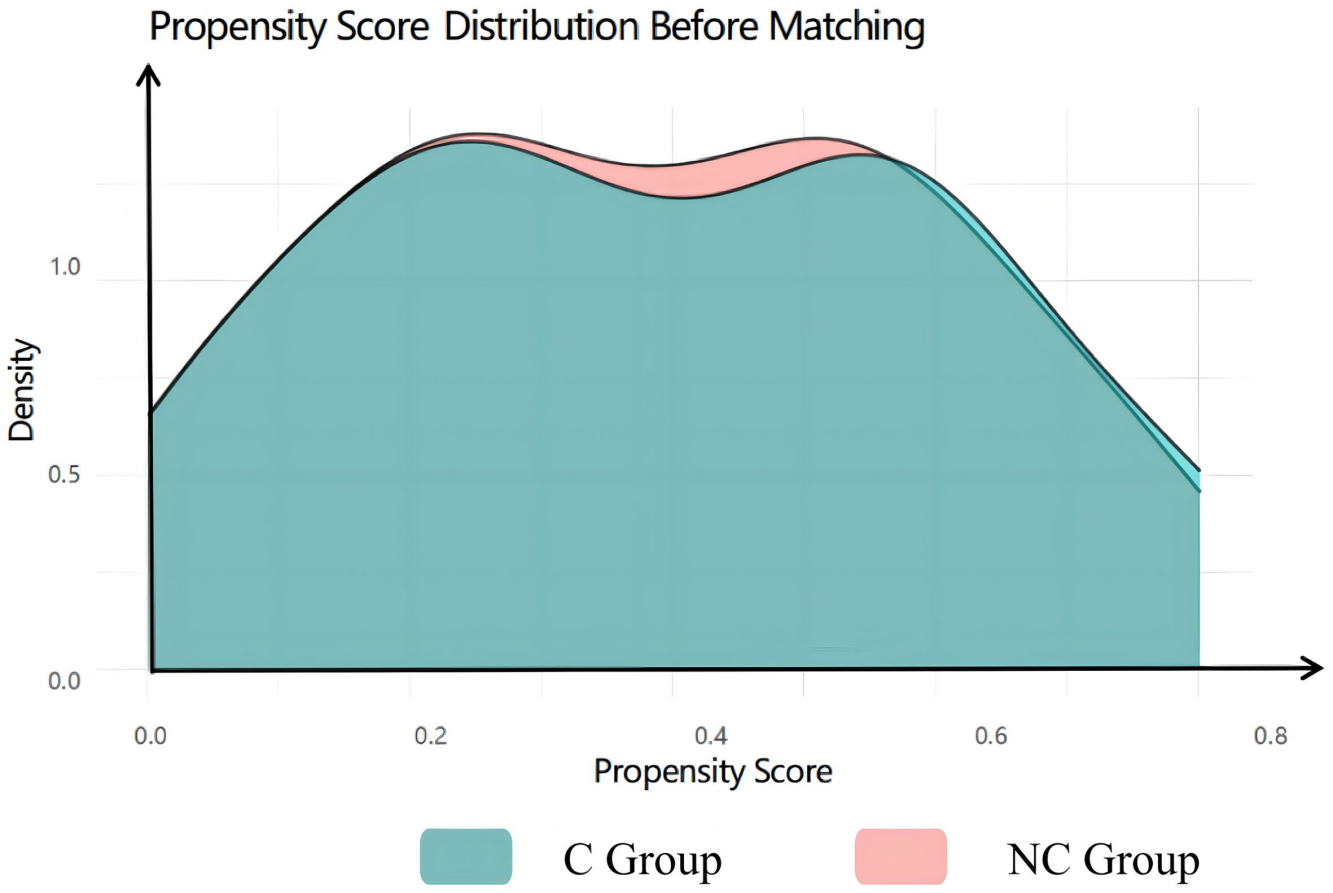
PS before matching.

**Figure 3:**
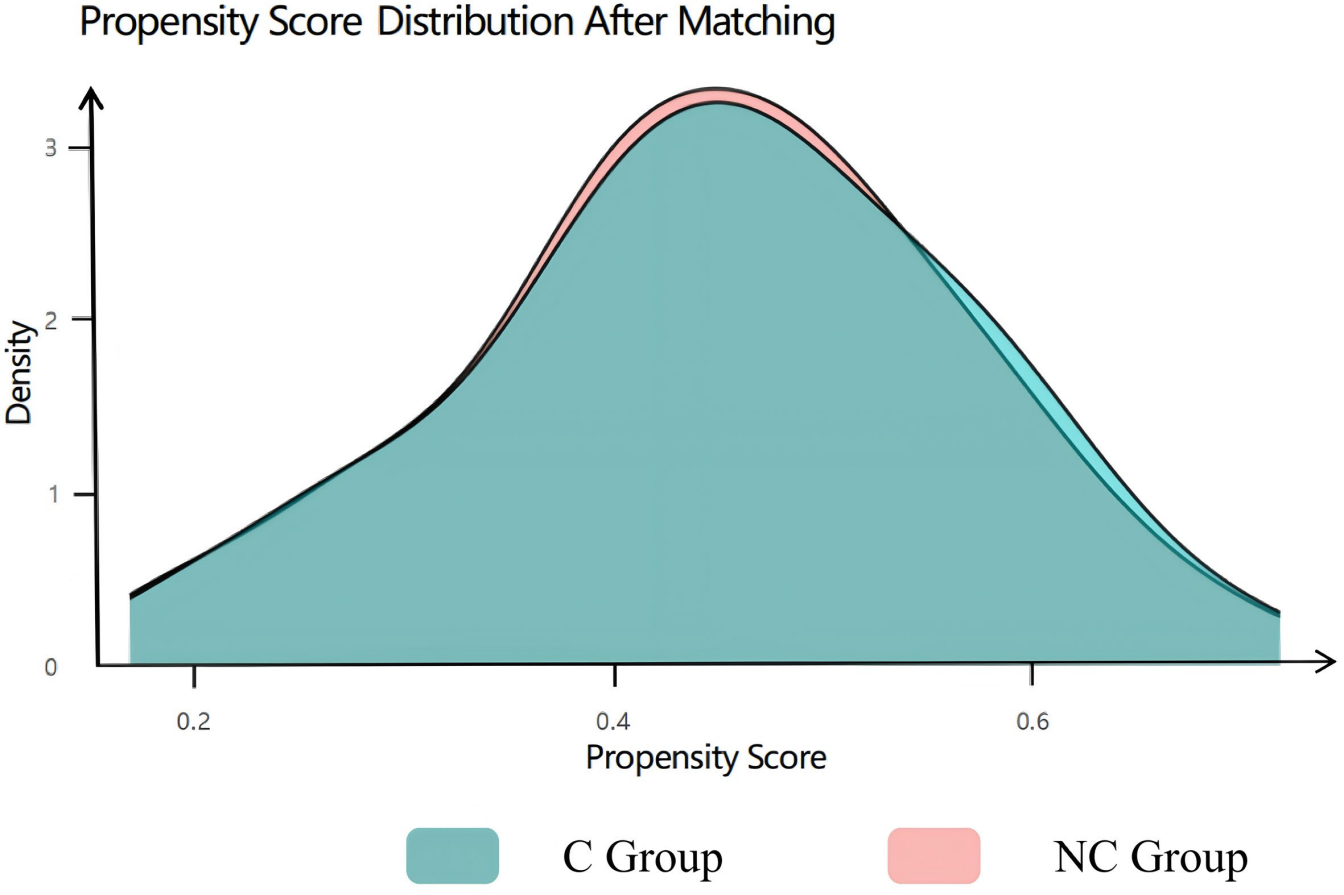
PS after matching.

**Figure 4:**
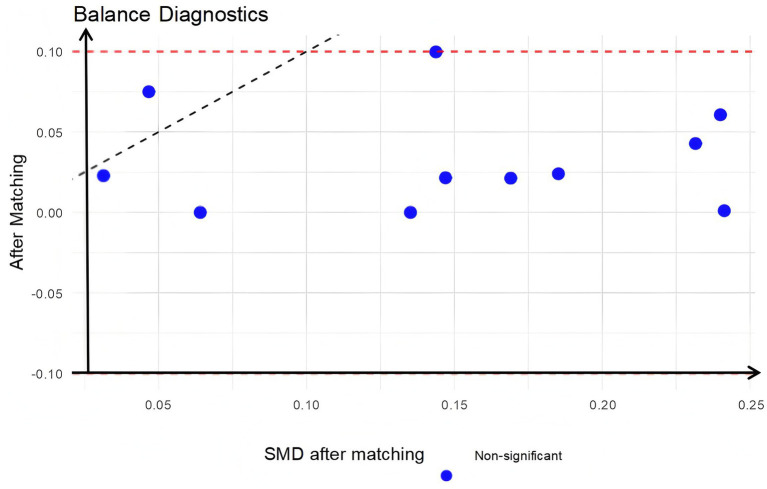
SMD after matching.

Visual inspection of propensity score distributions revealed substantial separation between groups before matching (Figure 2), which was effectively eliminated after PSM, with near-perfect overlap in the matched cohorts (Figure 3). Quantitatively, all standardized mean differences (SMDs) for covariates were <0.10 post-matching (Figure 4), indicating excellent balance. Consistent with this, the unmatched cohort exhibited modest imbalances in BMI and hypertension prevalence (Table 1). In contrast, Table 2 demonstrates that all baseline characteristics were well balanced between the matched groups, with no statistically significant differences observed (all *p* > 0.05).

**Table 1:** Comparison of basic information after PSM between the two groups.

Baseline	After PSM			
	Group C (*n* = 107)	Group NC (*n* = 107)	*t*/*χ*^2^	*p*
Age (year)	75.50 ± 8.68	75.92 ± 9.23	−0.342	0.733
BMI (kg/m^2^)	22.29 ± 2.64	22.2 ± 2.47	0.248	0.804
Years of education	2.44 ± 1.73	2.57 ± 1.29	−0.67	0.504
Man (*n*)	48	51	0.168	0.682
ASA (*n*)			0.936	0.333
II	48	41		
III	60	67		
Coronary heart disease (*n*)	22	26	0.429	0.513
Hypertension (*n*)	35	33	0.086	0.77
Diabetes mellitus (*n*)	26	24	0.104	0.747
Smoking (*n*)	25	29	0.395	0.53
Alcohol consumption (*n*)	26	30	0.386	0.535
Surgical site (*n*)			5.269	0.153
Thighbone	54	59	−0.342	0.733
Knee	32	20		
Tibia and fibula	17	18		
Foot and ankle	5	11		

**Table 2:** Comparison of basic information before PSM between the two groups.

Baseline	Before PSM			
	Group C (*n* = 120)	Group NC (*n* = 157)	*t*/*χ*^2^	*p*
Age (year)	76.08 ± 8.70	74.45 ± 8.83	−1.526	0.128
BMI (kg/m^2^)	22.41 ± 2.63	21.79 ± 2.48	1.997	0.047^*^
Years of education	2.38 ± 1.66	2.60 ± 1.32	1.168	0.244
Man (*n*)	55	77	0.281	0.596
ASA (*n*)			0.149	0.699
II	54	67		
III	66	90		
Coronary heart disease (*n*)	26	32	0.068	0.795
Hypertension (*n*)	43	39	3.944	0.047^*^
Diabetes mellitus (*n*)	33	28	3.7	0.054
Smoking (*n*)	26	44	1.456	0.228
Alcohol consumption (*n*)	27	47	1.921	0.166
Surgical site (*n*)			7.233	0.065
Thighbone	59	98		
Knee	35	26		
Tibia and fibula	18	21		
Foot and ankle	8	12		

### Perioperative clinical and sleep-related characteristics

As shown in Table 3, intraoperative management—including anesthesia duration, fluid administration, blood loss, anesthetic agent doses, and rates of transfusion or postoperative analgesia—was comparable between groups (all *p* > 0.05). However, Group C demonstrated significantly lower postoperative serum albumin (32.03 ± 4.75 vs. 34.36 ± 3.17 g/L; *p* < 0.001), higher CRP levels (80.79 ± 42.50 vs. 63.89 ± 28.73 mg/L; *p* = 0.001), greater pain intensity (VAS: 4.97 ± 1.54 vs. 4.28 ± 1.42; *p* < 0.001), and a higher proportion of procedures initiated during evening or night hours (20:00–8:00; *p* = 0.003).

**Table 3:** Comparison of clinical data after PSM between the two groups.

	Group C (*n* = 107)	Group NC (*n* = 107)	*t*/*χ*^2^	*p*
Postoperative albumin (g)	32.03 ± 4.75	34.36 ± 3.17	−4.222	<0.001^*^
Postoperative CRP mg/L	80.79 ± 42.50	63.89 ± 28.73	3.409	0.001^*^
Start time of anesthesia (*n*)			11.625	0.003^*^
8:00 ≤ *t* < 14:00	43	61		
14:00 ≤ *t* < 20:00	51	27		
20:00 ≤ *t* < 8:00	13	19		
Postoperative VAS	4.97 ± 1.54	4.28 ± 1.42	−3.417	<0.001^*^

Sleep- and circadian-related parameters are summarized in Table 4. Although total sleep duration did not differ pre- or postoperatively, Group C had an earlier preoperative sleep midpoint (1.64 ± 0.64 vs. 1.99 ± 0.78 a.m.; *p* < 0.001), suggesting a stronger morning chronotype at baseline. Postoperatively, these patients experienced more frequent iatrogenic sleep disruptions (6.99 ± 3.42 vs. 5.74 ± 3.66 events; *p* = 0.010), took longer daytime naps (125.05 ± 55.48 vs. 102.48 ± 58.04 min; *p* = 0.004), exhibited a larger phase shift in sleep timing (0.58 ± 0.97 vs. 0.22 ± 1.06 h; *p* = 0.012), and showed a more pronounced shift toward evening chronotype (*p* = 0.034). Group C had a significantly higher incidence of postoperative delirium (POD): 37 of 107 patients (34.6%) versus 20 of 107 (18.7%) in Group NC (*χ*^2^ = 6.911, *p* = 0.009). Additionally, hospital length of stay (LOS) was prolonged in Group C (13.56 ± 3.17 vs. 11.53 ± 2.72 days; *p* < 0.001) (Table 5).

**Table 4:** Comparison of sleep data after PSM between the two groups.

	Group C (*n* = 107)	Group NC (*n* = 107)	*t*/*χ*^2^	*p*
Preoperative sleep duration (min)	501.59 ± 69.25	500.19 ± 73.84	0.143	0.886
Postoperative sleep duration (min)	499.39 ± 92.89	518.41 ± 79.11	−1.612	0.108
Preoperative nap (min)	83.69 ± 45.70	76.40 ± 47.05	1.15	0.252
Postoperative nap (min)	125.05 ± 55.48	102.48 ± 58.04	2.908	0.004^*^
Preoperative sleep midpoint (am)	1.64 ± 0.64	1.99 ± 0.78	−3.561	<0.001^*^
Midpoint of sleep after surgery (am)	2.22 ± 0.70	2.21 ± 0.65	0.076	0.94
Point phase shift during sleep (min)	0.58 ± 0.97	0.22 ± 1.06	2.549	0.012^*^
Preoperative outdoor time (min)	133.88 ± 47.29	139.44 ± 109.56	−0.482	0.63
Postoperative outdoor time (min)	29.49 ± 32.03	34.86 ± 33.01	−1.209	0.228
Preoperative PQSI (*n*)	5.76 ± 2.36	5.62 ± 2.12	0.457	0.648
Postoperative PQSI (*n*)	8.66 ± 2.47	8.00 ± 2.63	1.902	0.058
Iatrogenic sleep disruption (*n*)	6.99 ± 3.42	5.74 ± 3.66	2.585	0.01^*^
Preoperative rhythm (*n*)			7.955	0.093
Absolute night type	6	13		
Moderate night type	20	14		
Intermediate type	26	32		
Moderate morning type	27	32		
Absolute morning type	28	16		
Postoperative rhythm (*n*)			10.398	0.034^*^
Absolute night type	6	13		
Moderate night type	25	14		
Intermediate type	24	32		
Moderate morning type	43	32		
Absolute morning type	9	16		

**Table 5:** Comparison of outcome data after PSM between the two groups.

	Group C (*n* = 107)	Group NC (*n* = 107)	*t*/*χ*^2^	*p*
POD (*n*)	37	20	6.911	0.009^*^
Length of hospital stay (*n*)	13.56 ± 3.17	11.53 ± 2.72	5.023	<0.001^*^

### Multivariable regression analyses

In multivariable logistic regression adjusting for anesthesia timing, VAS pain score, CRP, and albumin, perioperative circadian rhythm disruption remained independently associated with an increased risk of POD (adjusted OR = 2.26, 95% CI: 1.13–4.52; *p* = 0.022) (Table 6). Other independent predictors included nighttime anesthesia initiation (20:00–8:00; OR = 2.98, *p* = 0.021), greater iatrogenic sleep disruption (OR = 1.10 per event, *p* = 0.048), and higher postoperative albumin (OR = 1.11 per 1 g/L, *p* = 0.023). Concurrently, multiple linear regression confirmed that circadian rhythm change independently predicted a 1.026-day prolongation in LOS (*β* = 1.03, 95% CI: 0.25–1.80; *p* = 0.009), alongside lower albumin (*β* = −0.23, *p* < 0.001) and higher CRP (*β* = 0.012, *p* = 0.029) (Table 7). All variance inflation factors (VIFs) were <1.4, indicating negligible multicollinearity.

**Table 6:** Multifactor binary logistic regression of POD in two groups.

Exposure	*p*-value	95% LCI	OR	95% UCI	
Midpoint of sleep	0.229	0.432	0.726	1.222	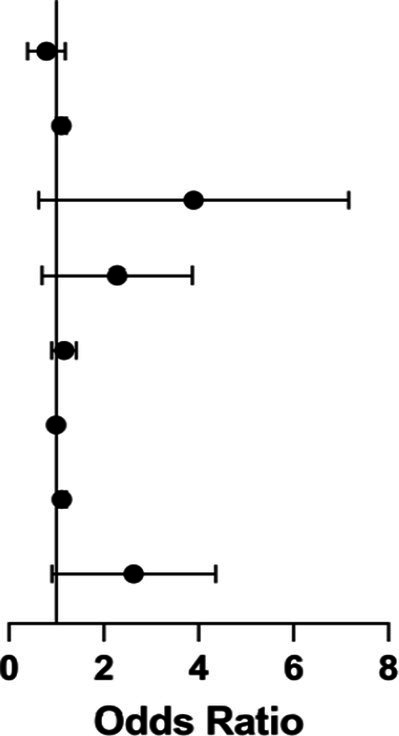
Iatrogenic sleep disruption	0.048^*^	1.001	1.103	1.215	
Start time of anesthesia (20:00 ≤ *t* < 8:00)	0.021^*^	1.183	2.984	7.527	
Start time of anesthesia (14:00 ≤ *t* < 20:00)	0.086	0.912	1.914	4.016	
Postoperative VAS	0.235	0.916	1.145	1.43	
Postoperative C-reactive protein	0.411	0.987	0.996	1.005	
Postoperative albumin	0.023^*^	1.015	1.113	1.222	
Circadian rhythm	0.022^*^	1.127	2.257	4.517	

**Table 7:** Multiple linear regression analysis of the length of hospital stay between the two groups.

Exposure	*β*	*t*	*p*	VIF	95% LCI	95% UCI
Circadian rhythm	1.026	2.621	0.009^*^	1.158	0.254	1.797
Iatrogenic sleep disruption	0.057	1.051	0.295	1.128	−0.050	0.163
Midpoint of sleep	−0.397	−1.422	0.157	1.263	−0.946	0.153
Postoperative VAS	0.095	0.746	0.457	1.13	−0.157	0.347
Postoperative albumin	−0.228	−4.513	<0.001^*^	1.354	−0.328	−0.129
Postoperative CRP	0.012	2.203	0.029^*^	1.149	0.001	0.022

### Subgroup analyses for effect modification

**Figure 5:**
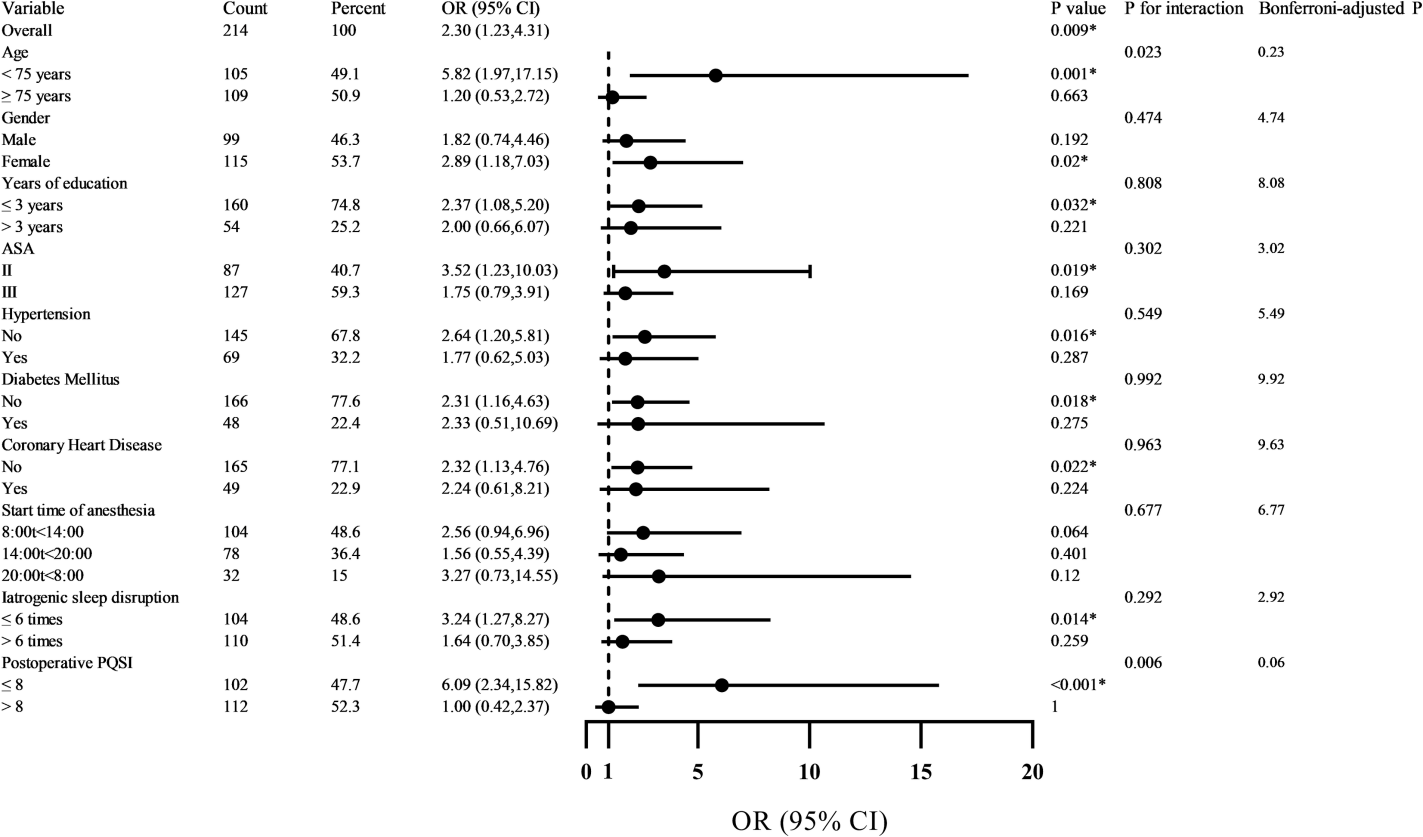
Forest plot of subgroup analysis on the impact of circadian rhythm change on POD.

Subgroup analyses explored whether the association between circadian rhythm disruption and POD varied across clinically relevant subgroups (Figure 5). The overall adjusted OR was 2.30 (95% CI: 1.23–4.31; *p* = 0.009). A statistically significant interaction was observed for age (*p* for interaction = 0.023): the effect was pronounced among patients <75 years (OR = 5.82, 95% CI: 1.97–17.15; *p* = 0.001) but absent in those ≥75 years (OR = 1.20, *p* = 0.663). Similarly, patients with better postoperative sleep quality (PSQI ≤8) exhibited markedly elevated risk (OR = 6.09, 95% CI: 2.34–15.82; *p* < 0.001), whereas no association was evident in those with PSQI >8 (OR = 1.00, *p* = 1.00); this interaction was also significant (*p* = 0.006). No other subgroups—including sex, education, hypertension, diabetes, coronary heart disease, or anesthesia timing—showed evidence of effect modification (all *p* for interaction >0.05). Given 10 prespecified subgroup comparisons, Bonferroni correction yielded an adjusted significance threshold of *α* = 0.005. While the interaction *p*-values (0.023 and 0.006) did not survive this correction, the magnitude and biological plausibility of the subgroup effects suggest potential heterogeneity in susceptibility to circadian disruption. These findings should be considered exploratory and require validation in future prospective studies.

## Discussion

Lower extremity orthopedic surgery—particularly for acute fractures in older adults—is accompanied by a constellation of perioperative stressors that profoundly disrupt circadian physiology. Severe postoperative pain activates the hypothalamic–pituitary–adrenal (HPA) axis and suppresses nocturnal melatonin secretion, thereby impairing sleep continuity and circadian alignment (16). Concurrently, restricted mobility limits exposure to natural daylight—the primary zeitgeber for the SCN—weakening entrainment of the central pacemaker (17). Compounding these effects, the hospital environment—characterized by nighttime lighting, frequent care interruptions, and irregular noise—further fragments sleep and desynchronizes peripheral oscillators in immune and metabolic tissues (18). Together, these factors may induce internal desynchrony between central and peripheral circadian clocks, a state implicated in neuroinflammation, impaired glymphatic clearance, and heightened vulnerability to POD (19).

Although circadian disruption is often viewed as a consequence of delirium, emerging evidence positions it as a modifiable upstream driver. Our prospective cohort study provides clinical support for this hypothesis: perioperative circadian rhythm change—quantified as a shift in scores on the MEQ-SA—was independently associated with a 2.26-fold increased risk of POD (adjusted OR = 2.26, 95% CI: 1.13–4.52; *p* = 0.022), even after rigorous adjustment via 1:1 propensity score matching. To our knowledge, this is among the first studies to apply the MEQ-SA to capture dynamic chronotype shifts in elderly patients undergoing lower extremity fracture repair and to demonstrate its predictive value for POD.

This association persists even when accounting for a broad array of clinical and procedural confounders in a multifactor binary logistic regression model. In this fully adjusted analysis, nighttime anesthesia initiation (20:00–08:00) emerged as the strongest independent predictor of POD, conferring nearly a threefold increase in odds (OR = 2.98, 95% CI: 1.18–7.53; *p* = 0.021). This finding reinforces the notion that imposing surgical stress during the biological night—when melatonin peaks and sleep drive is maximal—may critically destabilize circadian integrity in an already vulnerable population. In contrast, afternoon anesthesia (14:00–20:00) showed a non-significant trend toward elevated risk (OR = 1.91, *p* = 0.086), consistent with prior reports of suboptimal postoperative sleep following late-day procedures but lacking the same magnitude of circadian vulnerability (3). In older adults, whose SCN function is already compromised by age-related declines in rhythm amplitude and melatonin output, procedural stress during the biological night may amplify internal desynchrony. Nighttime procedures also coincide with essential but disruptive nursing activities, further perturbing circadian cues during peak sleep propensity. Thus, the timing of anesthesia relative to endogenous circadian phase may represent a clinically modifiable risk factor for POD (20).

Notably, iatrogenic sleep disruption—reflecting care-related interruptions such as nighttime vital checks or medication administration—was also independently associated with POD (OR = 1.10 per unit increase, 95% CI: 1.00–1.22; *p* = 0.048). This quantifies the tangible delirium risk posed by routine hospital practices that fragment nocturnal rest, supporting calls for “sleep-protective” care protocols. Conversely, neither postoperative pain intensity (VAS), systemic inflammation (C-reactive protein), nor intrinsic midpoint of sleep reached statistical significance in the multivariable model (*p* > 0.05 for all), suggesting their influence on delirium may be mediated through—or overshadowed by—circadian and temporal factors. For instance, while pain activates the HPA axis and suppresses melatonin (16), its direct contribution to POD appears attenuated once circadian misalignment and sleep fragmentation are accounted for.

These findings align with a growing body of translational evidence. Preclinical studies show that surgical stress under volatile anesthesia disrupts hippocampal clock gene expression and activates neuroinflammatory pathways, leading to delirium-like behaviors in aged mice—effects attenuated by melatonin supplementation (21). Clinically, Song et al. (22) reported that prolonged anesthesia duration exacerbates circadian misalignment and increases POD risk. Mechanistically, circadian disruption may impair cognitive recovery by dysregulating key neurotransmitter systems (e.g., acetylcholine, dopamine), blunting diurnal cortisol rhythms, and fragmenting restorative slow-wave sleep—all critical for maintaining cognitive stability (23, 24). Notably, melatonin levels plummet on the first postoperative day in patients receiving general anesthesia, potentially removing a key neuroprotective signal during a vulnerable neurobiological window (25).

Stratified analyses revealed compelling effect modification by age and postoperative sleep quality, as assessed by the PSQI. Circadian disruption conferred a markedly elevated POD risk in patients <75 years (adjusted OR = 5.82, 95% CI: 1.97–17.15; *p* = 0.001) but not in those ≥75 years (OR = 1.20, 95% CI: 0.53–2.72; *p* = 0.663; *p* for interaction = 0.023). This pattern may reflect differential circadian resilience: younger older adults likely retain greater baseline rhythm amplitude and neurobiological responsiveness, rendering them more susceptible to acute perturbations. In contrast, the oldest-old may exhibit a “floor effect,” wherein chronic circadian attenuation leaves little physiological reserve for additional disruption to manifest as clinical delirium (26, 27).

Equally notable was the interaction with postoperative sleep quality. Among patients with relatively preserved sleep (PSQI ≤8), circadian disruption strongly predicted POD (OR = 6.09, 95% CI: 2.34–15.82; *p* < 0.001), whereas no association was observed in those with poor sleep (PSQI >8; OR = 1.00, 95% CI: 0.42–2.37; *p* = 1.00; *p* for interaction = 0.006). This seemingly paradoxical result suggests that individuals with intact sleep architecture remain more sensitive to circadian rhythm change—possibly because their regulatory systems are still capable of generating robust physiological responses, which become maladaptive when temporal order is lost (28). Conversely, those with chronically poor sleep may already operate under maximal homeostatic stress, diminishing the incremental impact of acute rhythm change. Although these interactions did not survive Bonferroni correction for multiple comparisons (adjusted *α* = 0.005; yielding corrected *p* = 0.23 for age and 0.06 for PSQI), the consistency in direction, magnitude, and biological plausibility supports their potential relevance and warrants validation in future studies (29, 30).

We further examined the association between perioperative rhythm change and LOS. After adjustment for relevant clinical and demographic confounders, greater circadian —quantified as a per-unit change in MEQ-SA score—was independently associated with a clinically meaningful 0.94-day increase in LOS (*β* = 0.94, 95% CI: 0.16–1.72; *p* = 0.019). In older adults undergoing lower extremity orthopedic surgery, prolonged hospitalization is not merely a logistical or economic burden but a critical indicator of suboptimal recovery, reflecting delayed functional restoration, heightened susceptibility to hospital-acquired complications (e.g., infections, falls, deconditioning), and inefficient utilization of finite healthcare resources (31).

Notably, it was circadian rhythm change—not isolated sleep fragmentation—that emerged as a significant predictor of extended LOS. This distinction underscores that the adverse effects of temporal disorganization extend beyond sleep architecture alone (32). Rather, systemic desynchrony between central and peripheral clocks appears to impair core physiological processes essential for postoperative recovery, including immune surveillance, metabolic homeostasis, and tissue repair. These findings position circadian integrity not only as a neurocognitive safeguard against delirium but also as a broader determinant of surgical recovery trajectory.

Many drivers of circadian disruption—such as nighttime light exposure, non-urgent nocturnal care activities, and mistimed analgesia—are modifiable within the hospital environment (10). Targeted, low-cost interventions to preserve circadian alignment—such as structured light–dark cycles, consolidated nighttime nursing protocols, or timed melatonin administration—therefore represent a scalable strategy to enhance perioperative resilience. Such approaches hold dual promise: simultaneously mitigating neurocognitive risk (e.g., POD) and improving operational efficiency through shorter, safer hospital stays in this high-risk geriatric population.

### Limitations

(1) Although we adjusted for multiple confounders using multivariable regression and propensity score matching, residual confounding remains possible due to the non-randomized, observational nature of this study. (2) This research is a single-center study. Future research should consider multi-center, expanded sample size and extended observation time to enhance the credibility of the research results.

## Conclusion

In conclusion, our study identifies perioperative circadian rhythm change—as reflected by shifts in MEQ-SA scores—as an independent risk factor for both POD and prolonged hospitalization in older adults undergoing lower extremity orthopedic surgery. These findings position circadian stability as a novel, actionable target for perioperative neuroprotection. Future interventions—such as timed bright-light exposure, perioperative melatonin administration, or scheduling non-urgent procedures during the biological morning—may enhance circadian resilience and improve outcomes in this vulnerable population.

## Data Availability

The raw data supporting the conclusions of this article will be made available by the authors, without undue reservation.
